# Using High-Fidelity Virtual Reality for Mass-Casualty Incident Training by First Responders – A Systematic Review of the Literature

**DOI:** 10.1017/S1049023X24000049

**Published:** 2024-02

**Authors:** Sara Heldring, Maria Jirwe, Jonas Wihlborg, Lukas Berg, Veronica Lindström

**Affiliations:** 1. Department of Health Promoting Science, Sophiahemmet University, Stockholm, Sweden; 2. Falck Ambulance Sweden, Stockholm, Sweden; 3. Department of Health Sciences, Swedish Red Cross University, Stockholm, Sweden; 4. School of Health and Welfare, Dalarna University, Falun, Sweden; 5. Samariten Ambulance, Stockholm, Sweden; 6. Department of Nursing, Umeå University, Umeå, Sweden

**Keywords:** disaster medicine, Emergency Medical Services, high-fidelity simulation, mass-casualty incident, review, simulation training, situated cognition theory, virtual reality

## Abstract

**Introduction:**

First responders’ training and learning regarding how to handle a mass-casualty incident (MCI) is traditionally based on reading and/or training through computer-based scenarios, or sometimes through live simulations with actors. First responders should practice in realistic environments to narrow the theory-practice gap, and the possibility of repeating the training is important for learning. High-fidelity virtual reality (VR) is a promising tool to use for realistic and repeatable simulation training, but it needs to be further evaluated. The aim of this literature review was to provide a comprehensive description of the use of high-fidelity VR for MCI training by first responders.

**Methods:**

A systematic integrative literature review was used according to Whittemore and Knafl’s descriptions. Databases investigated were PubMed, CINAHL Complete, Academic Search Ultimate, Web of Science, and ERIC to find papers addressing the targeted outcome. The electronic search strategy identified 797 potential studies. Seventeen studies were deemed eligible for final inclusion.

**Results:**

Training with VR enables repetition in a way not possible with live simulation, and the realism is similar, yet not as stressful. Virtual reality offers a cost-effective and safe learning environment. The usability of VR depends on the level of immersion, the technology being error-free, and the ease of use.

**Conclusions:**

This integrative review shows that high-fidelity VR training should not rule out live simulation, but rather serve as a complement. First responders became more confident and prepared for real-life MCIs after training with high-fidelity VR, but efforts should be made to solve the technical issues found in this review to further improve the usability.

## Introduction

There is a need to optimize training and learning for emergency professionals to work safely and effectively in disaster situations. This systematic review focuses on first responders (the emergency professionals first entering the scene of a disaster) and their training for disasters with mass casualties. The often-heavy workload of a first responder in a mass-casualty incident (MCI) includes assessment and treatment of the injured, but also overall decision making on how to allocate available resources.^1,2^ Since MCIs are not frequent, in consequence, the first responders do not have the opportunity in their daily work to get experienced in managing these high-demand events.^1^

Today, efforts aiming to increase first responders’ experience and preparedness to manage an MCI include the development of checklists, training, and learning by reading or computer-based scenarios, and sometimes through live simulations with actors. Live simulations are known to be good for training and learning about disasters,^3^ but they are expensive, and many human resources are required to execute them; thus, they are rare.

When learning by reading or training in an environment that differs vastly from real life, the first responder might be unable to transfer the acquired knowledge into practice; in other words, there is a theory-practice gap.^4^ According to the theory of situated cognition,^4^ using high-fidelity simulations when training can reduce the theory-practice gap. The situated cognition theory originates from the constructivist theory of learning through experience and promotes clinical competency while strengthening clinical reasoning and reflective thinking skills,^4^ which are important competencies for first responders.^5^ When Kolb^6^ explains experiential learning, simulation training is strengthened by the notion that the learner experiences and practices in realistic environments. High-fidelity simulation with realistic scenarios where the learner is exposed to stressful situations is known to develop knowledge, skills, and experience.^7^ However, even if high-fidelity simulation is found to create good learning conditions,^3,7^ the possibility of repeating the simulation is crucial for learning.^7–9^

In recent years, new and innovative ways to enable high-fidelity and easily repeatable simulation with virtual reality (VR) have been developed. The use of technical solutions like VR for high-fidelity simulation, and the development of artificial intelligence (AI), have explosively increased during and after the coronavirus disease 2019 (COVID-19) pandemic, with seemingly good learning outcomes,^7^ and opens up for new creative possibilities for training.

To involve social, cultural, and physical interactions, it is important to facilitate cognitive, affective, and psychomotor learning.^10–12^ All these types of interaction can take place in a high-fidelity VR simulation, and AI enables the learners to interact with eventual avatars, depending on how the scenarios are created and programmed.

Simulation training with VR also provides the possibility to record and review scenarios, which theoretically allows self-correction. This can help first responders to construct their own learning as opposed to passively taken in and built upon pre-existing knowledge,^6^ which can support a shift from instruction to person-centered learning.^13^ The educator can become a facilitator, with a mission to provide feedback to encourage the first responder to reflect on their performance, which has several pedagogical advantages.^4^ In addition, by recording the scenarios, objective data can be extracted and used for systematic evaluation (eg, actions taken, time-effectiveness, communication, and clinical reasoning).

However, when searching in the literature, the definition of VR is broad. Some use the term VR when describing computer-based scenarios with a normal 2D screen, steered with a joystick or arrows on the keyboard.The term VR can also be used for more advanced 3D high-fidelity technology, for example with sensors in the hands and an immersive head-mounted display, or a fully immersive environment enclosed by four walls and 3D computer-based imaging that allows the user to step into a virtual world and interact with it. Due to the difference between 2D and 3D, it is not suitable (or possible) to compare them. Therefore, in this review, only the use of a 3D VR technique enabling high-fidelity simulation is included.

Introducing new and innovative ways to enable high-fidelity and repeatable simulation with VR can be seen as a suitable solution for training and learning for MCIs, but it is necessary to synthesize existing knowledge and to identify advantages and disadvantages before implementing it widely in educational and clinical contexts. Therefore, this literature review aimed to provide a comprehensive description of the use of high-fidelity VR for MCI training by first responders.

## Methods

As the research area concerning high-fidelity VR and MCI training by first responders is currently the subject of only a limited number of studies, an integrative systematic literature review was used to include findings from diverse research designs. Integrative reviews provide a range of perspectives and enable a more comprehensive understanding of the topic investigated, as opposed to only including studies using one research design.^14^ This integrative review was conducted in line with the standards of the Preferred Reporting Items for Systematic Reviews and Meta-Analyses (PRISMA) statement (PRISMA Checklist included as Supplemental Material; available online only).^15^

### Literature Search Strategies

The literature search was finished on March 23, 2023. Databases included were: PubMed (National Center for Biotechnology Information, National Institutes of Health; Bethesda, Maryland USA); CINAHL Complete (EBSCO Information Services; Ipswich, Massachusetts USA); Academic Search Ultimate (EBSCO Information Services; Ipswich, Massachusetts USA); Web of Science (Clarivate Analytics; London, United Kingdom); and ERIC (Institute of Education Sciences; Washington, DC USA). A series of comprehensive searches was carried out combining subject searches and free searches. The following keywords were chosen based on the researcher’s background knowledge of the topic and with support from a university librarian: *virtual reality, VR, simulated environment, virtual learning environment, virtual world, 3d environment, immersive simulation* AND *disaster preparedness, disaster victims, disaster planning, mass casualty incidents, disaster medicine, triage, mass casualties*. In addition, manual searches of the included articles’ reference lists were conducted, which led to the inclusion of one more article in the review.

**Figure 1.**
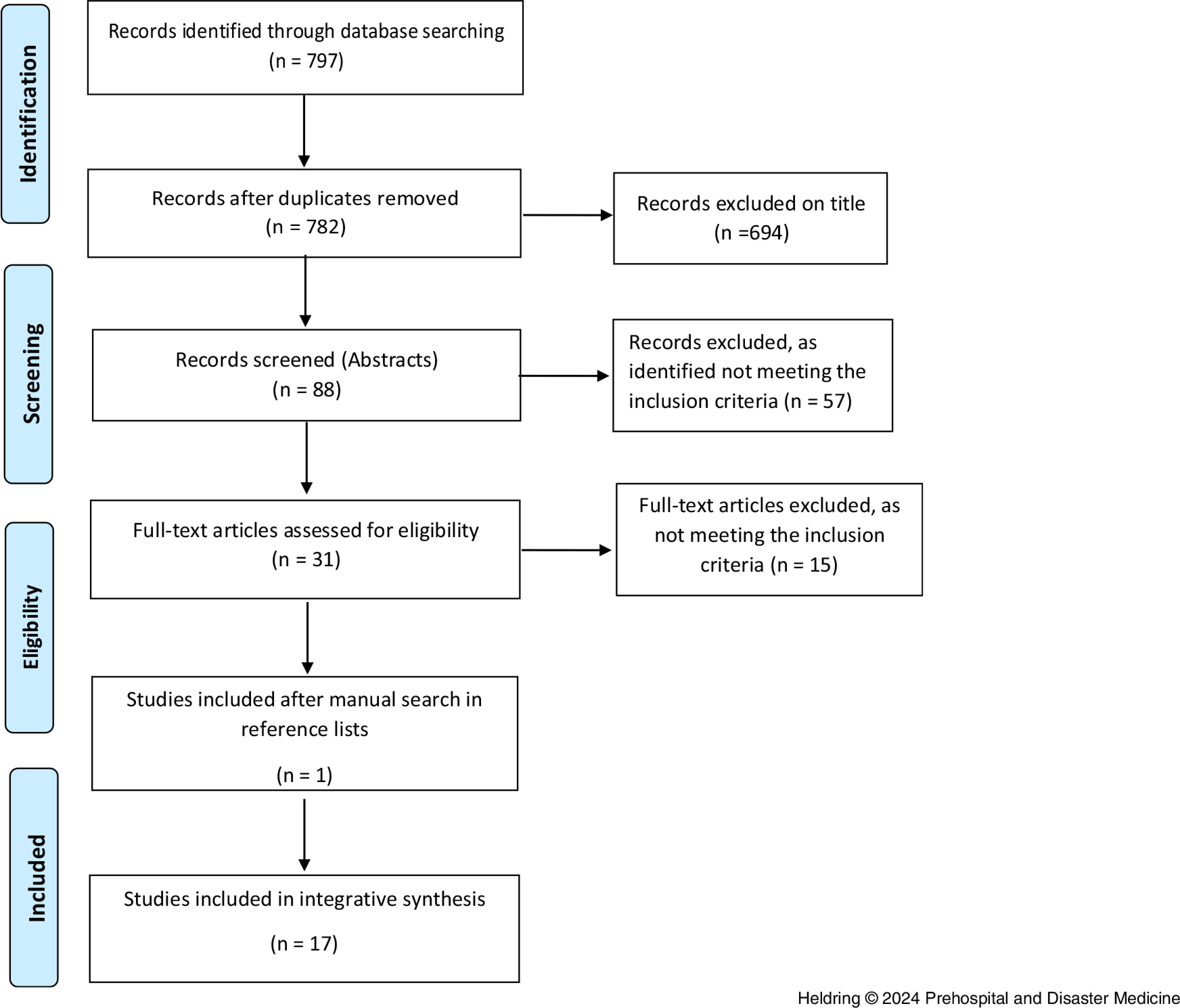
PRISMA Flow Diagram.

The inclusion criteria were: (1) peer-reviewed studies; (2) studies focusing on high-fidelity/3D VR; and (3) written in English. Exclusion criteria were: (1) studies focusing on 2D VR; (2) studies conducted in intra-hospital settings; and (3) studies older than 15 years, due to the area’s rapid technological development. The search strategy identified 797 potential studies, as shown in Figure 1. The titles were screened independently by the first author to the inclusion/exclusion criteria, and uncertainty was discussed with the other authors until a consensus was reached. In total, 88 abstracts remained after removing duplicates, and the included abstracts were then screened independently by the first and last author to the inclusion/exclusion criteria. Recurring meetings took place to discuss the process, and thus strengthen reliability throughout the selection process. The screening of abstracts resulted in the inclusion of 31 studies. The studies were scrutinized and discussed by the authors until consensus on inclusion was reached. In total, 17 studies were deemed eligible for final inclusion (Figure 1).

### Data Evaluation

Authenticity, methodological quality, and data relevance were considered in the evaluation process^14^ independently by the first and the last authors. The included studies were evaluated according to methodological rigor and risk of bias on a three-level scale (high, moderate, or low) following a checklist usable for both quantitative and qualitative research,^16^ and to the relevance of the research question on a two-level scale (high or low); Table 1.^14^ None of the studies were excluded based on this evaluation.

**Table 1.** Included Studies Matrix

Study Title, Citation, Location, Journal Name	Aim and Objectives	Research Design Data Collection	Sample Characteristics	Type of VR	Major Findings Relevant to the Review	Methodological Rigor (3-Level Scale)	Data Relevance (2-Level Scale)
“Comparative study of a simulated incident with multiple victims and immersive virtual reality” (Ferrandini, et al - 2018) Spain Nurse Education Today	To determine the efficiency in performing START triage, comparing VR to clinical simulation in an MCI. The secondary objective is to determine the stress produced in the health professionals in the two situations described.	Quantitative (Case-Control) Video Recordings Stress levels measured through salivary sampling.	Emergency Master’s Degree Nursing Students (n = 67)	VR with sensors and a fully immersive head-mounted display.	The percentage of correctly triaged victims was similar in both groups. The stress levels in salivary α-amylase were significantly higher in the live simulation group than in the VR group. The number of correctly triaged victims was not related to the participants’ level of stress or previous physical condition, or previous experience. Heart rate and blood pressure increased during the training but there were no differences between groups.	Moderate	High
“Decontamination training: with and without virtual reality simulation” (Farra, et al - 2015) USA Advanced Emergency Nursing Journal	To examine the use of VR simulation to teach the disaster-specific skill of decontamination.	Quantitative (Case-Control) Questionnaire Observations	Senior Nursing Students (n = 106)	VR with a gaming control capable of tracking participants’ full-body movements with a depth-sensing camera.	The VR group had better improvement in self-efficacy related to overall disaster skills than the control group. There was no difference between the groups in cognitive knowledge. The VR group did it in a significantly shorter time.	Moderate	High
“Virtual reality triage training can provide comparable simulation efficacy for paramedicine students compared to live simulation-based scenarios” (Mills, et al - 2019) Australia Prehospital Emergency Care	To compare the simulation efficacy of a bespoke VR MCI triage training simulation against a comparable live simulation scenario.	Mixed Methods Video Recordings Questionnaire Focus Group Interviews	Bachelor of Paramedical Science Students (n = 29)	VR with sensors and a fully immersive head-mounted display.	The heart rate and the perceived workload were significantly higher in the live simulation group than in the VR group. There were no differences regarding the percentage of correctly triaged victims, mental demand, demand, performance, effort, or frustration between the groups. A higher satisfaction with the objectives and information, support, problem-solving, feedback and fidelity provided was found in the VR group. The VR experience was comparable to live simulation in realism. This was true graphically but also the visual and auditory information was realistic. The VR simulation was described to be a suitable way to focus on and practice skills before adding more stress factors, as in live simulations. Using VR is cost-effective once VR content is developed.	High	High
“Virtual reality triage training provides a viable solution for disaster preparedness” (Andreatta, et al - 2010) USA Science of Simulation in Emergency Medicine	To determine if a fully immersive VR disaster drill is as effective as a comparable live disaster drill using SPs in teaching and assessing START triage knowledge and skills for EM residents.	Quantitative (Randomized Controlled Trial) Questionnaire Triage Score and Triage Rating	Students in Emergency Medicine (n = 15)	A fully immersive VR environment enclosed by four walls and 3-D computer-based imaging.	The triage performance rating was better in the VR group, and the number of correctly triaged patients. The post-test knowledge was better in the live simulation group.	Moderate	High
“Fireground cue recognition: effects on firefighter situational awareness when facing high-risk situations in virtual reality” (Bayouth, et al - 2019) USA International Fire Service Journal of Leadership and Management	To determine if situational awareness achieved via cue recognition and prior exposure to similar fire ground conditions reduce the potential risks for firefighters and victims in high-risk environments.	Quantitative Computer Measures Video Recordings Questionnaire	Firefighters (n = 62)	A fully immersive VR environment enclosed by four walls and 3-D computer-based imaging.	Cue recognition in VR training can lead to situational awareness. In VR, it is possible to get enough relevant cues to identify risk situations. Previous experience of risk situations made it more difficult to identify risk situations in the VR scenario.	Moderate	High
“Teaching mass casualty triage skills using immersive three-dimensional virtual reality” (Vincent, et al - 2008) USA Academic Emergency Medicine	To examine if learners improve in speed, accuracy, and self-efficacy when using VR.	Quantitative (Observational) Repeated-Measures Model Comparing Task Completion Questionnaire	Medical Students (n = 24)	VR with sensors and fully immersive head-mounted display.	When exposing participants to three sequential VR scenarios, they improved in speed and accuracy of triage for every scenario, but the highest improvement was between the first and the second scenarios. The participants became more confident that they were considered effective first responders and that they could prioritize resources and identify high-risk patients over time. The participants rated the VR simulation high and relevant for them as health care providers. The tempo and degree of difficulty were just right.	Moderate	High
“The student experience using virtual reality simulation to teach decontamination” (Ulrich, et al - 2014) USA Clinical Simulation in Nursing	To explore the student experience using VR simulation.	Qualitative Focus Group Semi-Structured Interviews	Senior Baccalaureate Nursing Students (n = 23)	VR with a gaming control capable of tracking participants full-body movements.	The participants described VR as an effective learning activity and felt engaged, involved, and immersed when experiencing VR. The participants felt safe when using VR; felt they could make mistakes without hurting the patient, and not dangerous for their personal security. The possibility to interact with the body in the VR training made it easier to remember the training when combining VR with other learning activities, such as lectures and demonstrations on manikins, enhancing the long-term memory. Technical issues caused frustration.	Moderate	High
“Using immersive simulation for training first responders for mass casualty incidents” (Wilkerson, et al - 2008) USA Academic Emergency Medicine	To explore the utility of immersive VR simulation for training first responders in a terrorism disaster scenario.	Qualitative Observations Video Recordings Interviews	Paramedics with at least Four Years of Experience (n = 12)	A fully immersive VR environment enclosed by four walls and 3-D computer-based imaging.	Participants showed enthusiasm about VR training and especially pointed out the realism and the possibility of repeating the training several times. The participants had problems with the first responder tasks when entering the scene of a disaster and they also had problems identifying security issues. The VR training raised anxiety levels which led to the high intensity of the experience. Assessing the avatars was more difficult than expected. Participants claimed they could never practice enough for these high-demand situations, but with VR this could be done more frequently. One of the most important benefits of VR training was the ease of adding variations to the scenarios. Weaknesses with VR training: initial motion sickness, and real-life scenarios would have involved more people from the crowd to interact with, making the work even more stressful.	Moderate	High
“Performance evaluation of AR/VR training technologies for EMS first responders” (Koutitas, et al - 2020) USA Virtual Reality	To examine which technology can best provide improved skills for first responders.	Mixed Methods Recorded Tapes, Questionnaires, and Self-Reported Surveys	EMS First Responders (n = 30)	VR with sensors and a fully immersive head-mounted display.	Participants improved their overall performance when training with VR. The improvement in muscle and cognitive memory was associated with the number of repetitions. Most of the participants preferred VR training over traditional training and highlighted that having completed the VR training, they now felt more prepared and confident about deployment.	Moderate	High
“360 virtual reality pediatric mass casualty incident: a cross-sectional observational study of triage and out-of-hospital intervention accuracy at a national conference” (Lowe, et al - 2020) USA Journal of the American College of Emergency Physicians Open	To explore the feasibility of using a 360 VR experience for the assessment of adolescent disaster readiness and to assess its acceptance among users.	Quantitative, (Cross-Sectional) Computer Measures and Questionnaire	Physicians (n = 162) Medical Students (n = 13) EMTs (n = 4) Others (n = 28)	Two different types of VR with sensors and fully immersive head-mounted display.	Two different types of VR were used, and no differences were found in total scores between the two. The participants stated that training with VR was engaging and enjoyable. The VR training made them feel more prepared for MCI. The participants stated that training with VR was more immersive than mannequin-based simulation training.	Moderate	High
“Effectiveness of two varying levels of VRS” (Smith, et al - 2018) USA Nursing Education Perspectives	To evaluate two types of VR with varying capabilities in comparison to the use of written instructions to teach the skill of decontamination to nursing students.	Quantitative (Quasi-Experimental) Questionnaire Observations	Nursing Students (n = 197)	VR with sensors and fully immersive head-mounted display, compared to VR on a 2D personal computer monitor with mouse and keyboard controls.	Overall changes in cognitive test scores and performance scores were significant over time. Both groups were faster at six months than immediately after training. No significant differences between VR and control groups in cognitive test scores, performance scores, or performance time.	Moderate	High
“Learning and retention using VR in a decontamination simulation” (Smith, et al - 2018) USA Nursing Education Perspectives	To examine the longitudinal effects of VR on learning outcomes and retention.	Quantitative (Quasi-Experimental) Questionnaire Observations	Nursing Students (n = 108)	VR with sensors and a fully immersive head-mounted display.	Both the VR group and control groups showed improvement on the cognitive test from the pre-test to the post-test because of training, and their performance on the test remained high through the retention period. The control group performed better on the pre-test scores. Despite this difference, the VR group and control groups scored equally on the post-test. Overall, participants tended to do better immediately after training than when tested five months later. The VR group was significantly faster both immediately after and at five months following training.	Moderate	High
“The student experience with varying immersion levels of VR simulation” (Farra, et al - 2018) USA Nursing Education Perspectives	To provide new evidence on how varying levels of immersion are perceived by nursing students using disaster-based VR educational experiences.	Qualitative Focus Group Interviews	Nursing Students (n = 32)	VR with sensors and a fully immersive head-mounted display, compared to VR or 2D monitor with mouse and keyboard controls.	Active learning was evident with both levels of VR. VR supports different learning styles, such as visual learning and hands-on learning. The more immersive VR was viewed as more realistic. Participants from both levels of immersion identified barriers to use, including technology glitches in the simulation, and physical responses causing motion sickness and dizziness. Motion sickness from the head-mounted display could detract from the positive experience of VR. The participants stated that VR was good for memory retention. They felt higher self-confidence after training with VR.	Moderate	High
“Virtual reality-based medical education versus lecture-based method in teaching start triage lessons in emergency medical students: virtual reality in medical education” (Behmadi, et al - 2020) Iran Journal of Advances in Medical Education & Professionalism	To evaluate the effectiveness of learning through virtual simulation in comparison with traditional method in learning of START triage knowledge and skill level.	Quantitative (Quasi-Experimental) Performance of a Course Exam and Self-Assessment Questionnaire	Emergency Medicine Students (n = 44)	Lecture-based training compared to virtual simulation-based training with LCD TV and a fully immersive head-mounted display.	The student’s rate of learning was measured by their scores at the end of the course exam in both groups. The mean scores of virtual simulation-based education were slightly higher than those of lecture-based education, but it was not statistically significant. The students were more satisfied with virtual simulation-based education than the lecture-based and the difference between the mean scores of satisfaction was statistically significant.	Moderate	High
“Exposure to a virtual reality mass-casualty simulation elicits a differential sympathetic response in medical trainees and attending physicians” (Tovar, et al - 2023) USA Prehospital and Disaster Medicine	To evaluate the changes in psychological and physiological stress of medical students and physicians at different stages of training.	Quantitative (Observational) Measurement of Changes in Heart Rate with ECG Cognitive Stress Measured using a Questionnaire	Physicians (n = 21) Medical Students (n = 14)	An MCI simulation was filmed with a 360º camera and shown to participants on a VR headset while simultaneously recording ECG.	Sympathetic nervous system activation was observed in all groups during the MCI compared to baseline and occurred independent of age, sex, years of experience, or prior MCI response experience. Overall, 23/35 subjects (65.7%) reported increased cognitive stress in the MCI. Resident and attending physicians had higher odds of discordance between SNS activation and cognitive stress compared to medical students.	Moderate	High
“The use of virtual reality to improve disaster preparedness among nursing students: a randomized study” (Shujuan, et al - 2022) China Journal of Nursing Education	To assess the impact of VR scenarios on disaster preparedness among nursing students.	Quantitative (Randomized Controlled Trial) Questionnaires and Simulation-Based Test Disaster Preparedness, Confidence, Performance Assessed at Baseline and End of Study	Nursing Students (n = 101)	VR with sensors and a fully immersive head-mounted display. A total of 12 highly interactive disaster nursing scenarios were used.	Results indicated the VR group significantly improved in disaster preparedness, confidence, and performance. After adjusting for age, gender, clinic experience, and VR experience, the true effect of VR was confirmed.	Moderate	High
“Effectiveness of the virtual reality chemical disaster training program in emergency nurses: a quasi-experimental study” (Chang, et al - 2022) Taiwan Nurse Education Today	To examine the effectiveness of the “VR chemical disaster training program” on disaster preparedness and self-efficacy among emergency nurses.	Quantitative (Quasi-Experimental) Two-Group Repeated Measures Self-Efficacy Questionnaire Both Groups took Pre-Test, Post-Test, and Delayed Test Three Weeks after Training	Emergency Nurses (n = 67) Participants in the experimental group were significantly younger and less experienced in disaster management than those in the control group	VR simulation training was developed using a 360° immersive camera with sensors. The experimental group received VR simulation training, while the control group received tabletop drills.	There were no between-group differences in the participants’ self-assessment of disaster preparedness and self-efficacy before the intervention. The intervention group showed significantly higher self-assessment in chemical disaster preparedness scores than the comparison group one week after the intervention. No significant differences were found three weeks after the intervention.	Moderate	High

Abbreviations: VR, virtual reality; MCI, mass-casualty incident; START, Simple Triage and Rapid Treatment; EM, Emergency Medicine; EMS, Emergency Medical Services; EMT, emergency medical technician; ECG, electrocardiogram.

### Data Analysis

The data analysis process followed Whittemore and Knafl’s methodology for integrative reviews^14^ and Patton’s descriptions of qualitative analysis.^17^ To get a thorough understanding of the included studies, each study was read several times. First, extracted data were compared item by item and similar data were coded, categorized, and grouped. The categories were compared to the use of VR for MCI training by first responders. Data were visualized in a spreadsheet. A data comparison was performed by the authors interacting in an iterative process to identify patterns to ensure trustworthiness of the analysis. The search for patterns and the condensation of codes resulted in two categories and eight subcategories.

### Characteristics of Studies

The included studies were conducted in the United States (n = 12), Australia (n = 1), Taiwan (n = 1), China (n = 1), Iran (n = 1), and Spain (n = 1). The number of participants in the included studies varied between 12 and 207 (n = 1,159). The participants had different professional backgrounds: nursing students (n = 634), paramedic and emergency medicine students (n = 88), medical students (n = 51), physicians (n = 183), professional firefighters (n = 62), professional paramedics/emergency medical technicians (n = 46), emergency nurses (n = 67), and others (n = 28). Studies using quantitative methods were in the majority (n = 12), but there were also three studies that used qualitative methods and two studies that used mixed methods.

### Ethical Issues

The study was conducted aiming for good ethical practice with transparency, accuracy, and avoidance of plagiarism.^18^ All included studies were viewed as aligned with the Declaration of Helsinki statement of ethical principles for medical research.^19^ The declaration has been considered throughout the whole process but did not lead to the exclusion of any studies.

## Results

The findings are presented in two categories answering the study aim: to describe the use of high-fidelity VR for MCI training by first responders. The two categories, Learning Aspects and Usability, included eight subcategories in total.

### Learning Aspects

This category consisted of several aspects of learning when using VR: self-experienced by first responders, their performance during training, and as post-test outcome after VR training. Effects on learning, effects on knowledge retention, physiological response, and improved preparedness were learning aspects found when using VR for MCI training.

*Effects on Learning*—First responders described VR as a suitable and effective learning activity that improved their performance.^20–24^ The VR training was described to be a suitable way to focus on and practice individual skills and problem-solving abilities.^24–26^ A common skill to practice when using VR for MCI training was the triage of victims. In total, there was a small preference for the VR type of training in the triage performance compared to live simulation.^20,21,25,27^ A described important benefit of using VR for training and learning was the easiness to repeat the training,^21,26,28^ to control and add variations to the scenarios,^26,28^ which also decreased some of the intimidating factors that first responders experienced with live-simulation training.^29^ Especially two factors made the first responders feel safe in the learning environment when training with VR: they could make mistakes without hurting the patient and they did not put themselves into danger.^28,30^ The VR training supported different learning styles, such as visual learning and hands-on learning.^28,29^ Active learning or being active in the learning process was evident on several immersion levels of VR.^29^ During training with VR, the first responders could get feedback on their performance as they proceeded through the simulation and thus become better aware of their own ability.^28,29^

*Effects on Knowledge Retention*—The way retention of knowledge was gained from training with VR was described as a positive outcome.^22,28,29,31^ The possibility to be physically active in the VR training made it easier to remember and recall the training, and the hands-on training made it easier to extend the learning to real-life situations.^22,28,29^ The VR training improved muscle and cognitive memory^22,28,29^ and improved performance both in accuracy and time-effectiveness over time,^21^ and the improvements of knowledge retention were associated with the number of repetitions.^21,22^ The first responders stated that the reason for the better knowledge retention was that VR enabled a combination of muscle memory and visual reinforcement.^28,29^ When combining VR with other learning activities, such as lectures and live simulations, the long-term knowledge retention was even better.^28^ No differences were found between VR and different types of control groups when assessing retention of knowledge with pre- and post-tests,^23,27,31,32^ except in one study where the post-test results were better in the live-simulation group^20^ and one other study with better scores in the VR group compared to lecture-based education.^33^ Overall, first responders tended to do better immediately after training than when tested five months later.^27^

*Physiological Response*—Realistic scenarios causing stress and a physiological response was described as beneficial for learning.^25,26^ When the VR training was realistic, it raised the anxiety levels and led to an intense experience of the learning environment.^25,26,34^ However, compared to live simulation, the perceived amount of physiological work was lower when training with VR.^25,35^ Stress hormone levels measured in saliva^35^ and the heart rate at the beginning of the training^25,34^ were significantly lower when VR training was compared with live-simulation training. However, both heart rate and blood pressure increased during the training scenarios, and in total, there were no differences between VR and live-simulation training.^35^ Nevertheless, the overall physiological demands were perceived higher with live simulation.^25,34^

*Improved Preparedness*—The first responders became more confident in their overall disaster skills after training with VR, and they felt more prepared for real-life MCIs.^22,24,26,27,29,31,36^ They highlighted that they felt more capable of prioritizing resources and identifying high-risk patients^21,30^ and felt they could gather enough relevant cues to identify risk situations in general after training.^30^ Being able to experience chaos and to make mistakes, and to have the mistakes identified and corrected, made the first responders feel more prepared,^26^ especially when they had the possibility to repeat the training and re-evaluate their own actions.^24,26,27^

### Usability

This category involved different aspects that could affect the usability of VR. The term “usability” describes both user experience and other aspects that influence how suitable VR is as a tool for training, and thereby affecting the use of VR for MCI training. Training with VR could be difficult without tutorials, and technical aspects also affected the VR experience. How realistic and true-to-life VR training is affects the level of user satisfaction.

*Tutorials*—Tutorials on how to use the VR tool were found to be important prior to scenario training.^25,28^ The first responders agreed that it did not take long to become familiar with the technique, but an introduction was useful to avoid pushing the wrong buttons, leading to incorrect results.^25^ Tutorials that took 10-15 minutes were deemed adequate before starting the VR training. It took less than five minutes to become comfortable enough with the VR technique and to be able to focus on the decision-making tasks in the scenario.^26^ First responders that played videogames when they were children stated that they felt comfortable learning how to use VR and that VR was suitable for their generation.^29^

*Technical Aspects*—Technical concerns were distracting and frustrating, and this was the subject that first responders had the most comments about regarding desired improvements.^28^ Examples of technical concerns could be that the screen kept freezing and that the VR program got stuck, making the first responder unable to move forward.^29^ Moreover, first responders who noticed motion sickness and dizziness from using the head-mounted display stated that this reduced the positive experience of VR.^29^ Some first responders had problems identifying safety issues in the VR environment and believed it had technical reasons.^26,30^ Another technical aspect was how cost-effective VR is; one study showed that cost neutrality appeared when there were 145 participants compared to the costs for live simulation.^25^

*Realism*—First responders considered VR comparable to live simulation in relation to realism.^25,26,29^ Training with VR was in some ways more immersive than live-simulation training, but no differences were found regarding mental demand, temporal demand, performance, effort, or frustration between the two training methods.^25^ Some things were perceived as problematic to make realistic with VR (eg, human interaction and emotional immersion),^25^ and it was described that real-life scenarios would have involved more people to interact with, making the workload even more stressful.^26^ However, some first responders described their ability to focus on MCI skills was facilitated when they were not disturbed by emotions.^25^ Assessing the avatars in the VR training was more difficult than expected, because of noise, radio distractions, and competing demands at the scene, which replicated the challenges of a real MCI.^26^ The more immersive level of VR, the more realistic.^29^ First responders suggested that all tasks you do in real-life scenarios should also be included in the VR training.^28^

*Satisfaction*—First responders preferred VR training over traditional training.^22,25,26,33^ Reasons described included satisfaction with the support, problem-solving ability, the possibility to add variations, and to get feedback,^25,26,28^ but the realism and the possibility to repeat the scenarios several times were prominent reasons.^21,25,26,28^ The first responders claimed that they could never practice enough for these high-demand events, but with VR, this could be done more frequently.^26^ They felt engaged, involved, and immersed when experiencing MCI training with VR.^21,27–29,36^ They also rated the VR simulation as relevant for them in their profession as first responders^21^ and wished for VR to be more integrated into medical education, especially for MCIs.^36^ According to the first responders in one study, both directed and structured learning and time for free play in the VR scenarios were desired.^28^ The first responders repeatedly described the importance of learning being fun, and stated that the quality of the VR was important to enhance this.^29^ One weakness with VR training that was pointed out as negative for satisfaction was that some first responders initially had motion sickness.^26^

## Discussion

To the authors’ knowledge, this is the first integrative review including only high-fidelity VR in the context of first responders’ training for MCIs. Since an integrative review methodology was used,^14^ including studies using different research designs, it was possible to get a comprehensive understanding with qualitative studies providing an in-depth understanding and quantitative studies providing statistical measures. The overall findings may be seen as giving a broadened understanding of first responders’ use of high-fidelity VR for MCI training. When interpreting the results in the discussion, the theory of situated cognition was used to strengthen the theoretical rigor.^4^

This integrative review confirms that high-fidelity VR is a promising tool to use for MCI training by first responders. The dominant finding is that using VR enables repetition in a way not possible with live simulation.^21,26,28,36^ The positive learning and knowledge retention when using VR for training in MCIs was prominent^22,25,26,28,29,32^ and learners preferred VR training over other types of training.^22,25,26^ In the light of the theory of situated cognition, the possibility to be physically active, to train and learn in a realistic environment, to add variations to the simulations, and to get computer-generated feedback could be some of the reasons behind the positive outcome. Also, the results promote VR training as a suitable way to focus on and practice individual skills and problem-solving abilities.

However, as first responders need to train in realistic environments to narrow the theory-practice gap,^4^ the continuous need for live simulation should not be under-estimated. Even if first responders felt that high-fidelity VR was comparable to live simulation in realism,^25,26,29^ physiological responses and stress levels were perceived to be higher with live simulation than with VR.^25,35^ The theory of situated cognition argues that psychomotor involvement is an important factor for learning.^4^ Hence, there is a risk that the first responders might be less able to transfer acquired knowledge from VR training into practice. If so, the use of VR should not rule out live simulation of MCIs, but rather should be seen as a complement. However, the effect on physiological response should be interpreted with caution, as VR technology is constantly developing and ought to be more immersive soon. When the VR technology improves, it could potentially play a larger part in stress management training to enable first responders to better manage MCI-related stressors and mitigate the negative effects of high-stress exposures.

In the wake of the COVID-19 pandemic, and the rapid development of AI, the interest in technical solutions for alternative teaching methods will probably increase the use of VR even more. The results of this review show that the more immersive level of VR, the more appreciated,^29^ which indicates better outcomes of VR with improved techniques. However, it is important to keep in mind that advanced technique is no guarantee of sufficient learning and knowledge retention. The VR training should include a pedagogically stringent curriculum reflective of its learning objectives to ensure positive learning outcomes.^37,38^ If the VR training is not well-planned, composed, and executed, the learner might not be able to learn – no matter how advanced and immersive the VR technique. Another concern about using VR is the technical problems users find distracting and frustrating.^28^ Furthermore, the motion sickness and dizziness that some first responders experienced when using the VR system^29^ could be a barrier to implementation. Efforts should be made to solve these technical issues to reduce usability problems. As the VR technique is improving, it will likely cost less in the future, even though this review indicates that VR is already relatively cost-effective once the VR program is developed.^25^ In this review, no studies considered the possibility for several first responders to simultaneously interact in the VR simulation, something that is technically possible today, which could enable more social and cultural learning opportunities, as emphasized in the theory of situated cognition.^4^

Another result in this review is that training with VR offers a safe learning environment.^28,30^ If first responders can make mistakes without hurting the patients, and without putting themselves in danger during training, this could potentially promote edgier decision making and enable courage to test creative solutions. More research is needed to further explore first responders’ decision making during MCIs, and if VR could be a suitable tool to use for systematic evaluation. The on-going technological developments and advancements with VR have the potential to provide and develop a more person-centered way of learning and training^13^ on how to handle MCIs, and this needs to be evaluated further. Finally, VR provides a viable research tool for examining MCI training, as well as a platform to test learning, knowledge retention, and accuracy of performance, and to compare different MCI systems. It also provides a flexible, consistent, on-demand training method that is both stable and repeatable and is a promising tool for systematic development of protocols and performance standards for MCIs in the future.

## Limitations

A limitation of this review is that there were no high-level methodological papers included. Moreover, one could argue about the difficulty of drawing conclusions based on the limited number of studies available. However, the research area is young, and with new and promising techniques that many are eager to implement, there is an urgent need to review existing knowledge and to identify new areas of interest for research and development.

## Conclusions

This integrative review shows that an argument for using VR for MCI training is that VR scenarios are easy to repeat. Training for MCIs with VR made first responders feel more confident and prepared for real-life MCIs. The realism that VR entails is like live simulations, but the physiological demands and stress levels are not. This disparity between real-life and VR can lead to first responders not being able to transfer their obtained knowledge into practice. If this limitation cannot be solved by better techniques, the use of VR should not fully replace live simulations, but rather should complement them. Training with VR means a safe and controlled learning environment where first responders can make mistakes, correct those mistakes in real-time, self-reflect, and learn from the mistakes without fear of compromising safety. Virtual reality can be more cost-effective than live-simulation training and efforts should be made to solve the technical issues found in this review to further improve the usability of VR for MCI training in the future.

## Supporting information

Heldring et al. supplementary material Heldring et al. supplementary material

## Supplementary Materials

To view supplementary material for this article, please visit https://doi.org/10.1017/S1049023X24000049
